# Identifying the Optimal Exercise Prescription for Patients with Coronary Artery Disease Undergoing Cardiac Rehabilitation: Protocol for a Systematic Review and Network Meta-Analysis of Randomized Control Trials

**DOI:** 10.3390/ijerph191912317

**Published:** 2022-09-28

**Authors:** Shraddha Shah, Grace Dibben, Aditi Ketkar, David L. Hare, Jonathan Myers, Barry Franklin, Abraham Samuel Babu, Rod S. Taylor

**Affiliations:** 1 Department of Physiotherapy, Manipal College of Health Professions, Manipal Academy of Higher Education, Manipal 576104, India; 2 MRC/CSO Social and Public Health Sciences Unit & Robertson Centre for Biostatistics, Institute of Health and Well Being, University of Glasgow, Glasgow G12 8QQ, UK; 3 DES Institutes, Brijlal Jindal College of Physiotherapy, Pune 411004, India; 4 Department of Cardiology, Austin Health, University of Melbourne, Melbourne, VIC 3084, Australia; 5 Department of Cardiology, Veterans Affairs Palo Alto Health Care System/Stanford University, Palo Alto, CA 94304, USA; 6 Department of Preventive Cardiology and Cardiac Rehabilitation, William Beaumont Hospital, Royal Oak, MI 48073, USA

**Keywords:** angina pectoris, cardiac rehabilitation, coronary artery disease, coronary artery by-pass graft, exercise training, network meta-analysis, percutaneous coronary intervention

## Abstract

Coronary artery disease (CAD) is one of the leading causes of mortality and morbidity. Exercise-based cardiac rehabilitation (EBCR) has been shown to improve clinical outcomes in these patients, and yet clinicians are often challenged to prescribe the most effective type of exercise training. Therefore, this systematic review and network meta-analysis (NMA) aims to formally quantify the optimal dose of exercise training interventions to improve exercise capacity and quality of life by undertaking direct and indirect pooled comparisons of randomized controlled trials. A detailed search will be conducted on PubMed/MEDLINE, Cumulative Index to Nursing and Allied Health (CINAHL), EMBASE and Web of Science. Two reviewers will screen the existing literature and assess the quality of the studies. Disagreements will be resolved through consensus. We anticipate that the analysis will include pairwise and Bayesian network meta-analyses. Most of the trials have studied the impact of exercise training comparing one or two modalities. As a result, little evidence exists to support which interventions will be most effective. The current NMA will address this gap in the literature and assist clinicians and cardiac rehabilitation specialists in making an informed decision. Results will be disseminated through peer-reviewed journals. Ethical approval is not applicable, as no research participants will be involved. PROSPERO Registration number: CRD42022262644.

## 1. Introduction

Coronary artery disease (CAD) is one of the leading causes of mortality worldwide [1]. With advances in coronary revascularization and secondary prevention interventions, including cardioprotective pharmacotherapies, survival in these patients has improved considerably [2,3]. Secondary prevention in the form of cardiac rehabilitation (CR) includes multiple core components that target varied risk factors contributing to recurrent cardiovascular events. Medically supervised and home-based exercise training remains at the center of this multi-disciplinary approach. Exercise-based cardiac rehabilitation (EBCR) and its beneficial effects are well established in patients with CAD [4]. CR has proven to improve physical performance, increase cardiovascular function, reduce mortality, reduce stress and anxiety, and improve quality of life [4,5,6,7]. Despite the proven clinical outcomes and cost-effectiveness of EBCR, it remains highly underutilized due to low CR referral, uptake, and adherence rates [5,8,9].

In tandem with a more comprehensive approach to rehabilitation that includes counseling and education on the risk factors, disease management, and psychosocial interventions, EBCR has evolved to include varied forms of exercise training, intensities, and doses. Several leading international organizations have established recommendations for exercise prescription, including the American Heart Association, the American College of Sports Medicine, the American Association of Cardiovascular Prevention and Rehabilitation, and the European Society of Cardiology [10,11,12,13].

The latest Cochrane systematic review and meta-analysis of randomized controlled trials have also demonstrated significant benefits of EBCR in patients with CAD, including improvements in exercise capacity, health-related quality of life (HRQoL), and reduced risk of hospital admission [4]. Despite strong substantiating evidence and authoritative international support, the practicing clinician is often faced with the challenge of prescribing safe and effective regimens that enhance long-term adherence and outcomes.

There are few head-to-head trials available in CR that compare different types of exercise, dose, intensity, and outcomes to inform practice. Network meta-analysis (NMA) provides a statistical approach that facilitates both direct and indirect comparisons to explore the comparative effectiveness of different interventions [14]. Based on an NMA of randomized controlled trials, the primary aim of this study is to determine the optimal exercise training interventions and their doses to improve long-term adherence, exercise capacity, and HRQoL in CAD. Secondary aims are to assess the impact on mortality and major adverse cardiovascular events, to explore whether significant age and gender differences exist between these exercise interventions, and to identify potential confounders.

Therefore, the objective of this review is to design and propose a protocol that compares the effectiveness of varied exercise-based training interventions in patients with CAD. This network meta-analysis will allow us to make multiple direct and indirect comparisons among the relevant studies reported to date to determine the optimal exercise-based interventions from several perspectives.

## 2. Methodology and Materials

This protocol was developed following the Preferred Reporting Items for Systematic Reviews and Meta-Analysis for protocol (PRISMA-P) and PRISMA for Network Meta-Analyses (PRISMA-NMA) checklists [15,16] and registered with PROSPERO (CRD42022262644).

### 2.1. Search Strategy and Information Sources

The search strategy was developed in collaboration with subject experts and based on previous Cochrane reviews [4]. The search strategy was built using the PICO format (Table 1).

The databases of PubMed/MEDLINE, Cumulative Index to Nursing and Allied Health (CINAHL), EMBASE, and Web of Science will be searched using combinations of selected MeSH terms and free text terms that are synonymous with exercise-based CR and CAD. The filters of human trials, English language, and randomized control trials (RCTs) will be applied to this search. Furthermore, to reflect contemporary medical practice, only studies published after 2000 will be included. Furthermore, the reference lists of the retrieved articles and systematic reviews will be searched manually. The search string developed for PubMed is detailed in Table 2. The detailed search for each individual database is given in Supplemental Table S1.

After completing the search, the retrieved articles will be compiled in a reference manager and duplicates will be removed. The screening of the title and abstracts will be conducted independently by two independent reviewers (SS/AK). A third person (ASB) will serve as an arbitrator. Once the titles and abstracts of the trials have been screened and duplicates are removed, the eligible full texts meeting the inclusion criteria will be used for data extraction. The reasons for exclusion will be tracked and reported in the PRISMA flow diagram.

### 2.2. Eligibility Criteria and Study Selection

For inclusion, studies must be published in English in a peer-reviewed journal. The inclusion of studies will follow the Participants, Intervention, Comparator, Outcomes, and Study design structure [16]. Study participants should include adults (>18 age) of either gender, who have documented CAD (i.e., angina, myocardial infarction; MI) with or without coronary revascularization (i.e., percutaneous coronary intervention; PCI with or without stenting, or coronary artery bypass grafting; CABG). Studies with heterogeneous populations from the aforementioned diagnoses will also be included if patients underwent EBCR as their primary intervention with or without adjunctive interventions. The type of exercise training and intensity will be determined by pre-specified definitions, as detailed in Table 3.

Studies are required to have at least one outcome that measures exercise capacity (i.e., peak oxygen consumption; peakVO_2_, or functional capacity from a six-minute walk test (6MWT) or changes in metabolic equivalents (METs) from a cycle or treadmill test or exercise time in minutes) either through any testing protocol or estimated METs from an incremental shuttle walk test (ISWT). Secondary outcomes related to acute cardiac events, HRQoL, mortality, and hospitalizations will also be collected. Inclusion will be limited to RCTs comparing any form of exercise training as their primary intervention to standard care or any other form of exercise-based intervention. Exercise-based interventions will include aerobic training, resistance training, high-intensity interval training, inspiratory muscle training, or combinations thereof. The studies comparing the interventions in heart failure patients will be excluded.

### 2.3. Data Extraction

Data from the selected studies will be extracted by two independent reviewers (SS and AK) and reported in a pre-specified data extraction Excel form that will include the following details: study (author, country, sample size, duration), participants (age, gender, diagnosis, medical/surgical intervention, comorbidities, duration since diagnosis), intervention (mode, frequency, intensity, time, type, adherence), comparator intervention, adverse events, primary and secondary outcomes, and results. These data will be subsequently entered into an electronic tool such as RevMan. Any discrepancies between the two authors will be resolved by the third reviewer (ASB) and the senior author (RT).

### 2.4. Risk of Bias

Two reviewers (SS and AK) will independently assess the risk of bias using the Cochrane Collaboration’s Risk of Bias 2 [17]. This tool assesses the studies under the following domains: selection bias (random sequence generation and allocation concealment), performance bias (blinding of patients and personnel), detection bias (blinding of outcome assessment), attrition bias (incomplete outcome data), reporting bias (selective outcome reporting) and other biases.

In case of disagreement, consensus will be reached through discussion and when not possible, a third reviewer (ASB) will be approached.

## 3. Data Synthesis and Analysis

The synthesis and analysis will be performed by GD, SS, and RT and will be specified for continuous and dichotomous measures. For continuous outcomes, data will be pooled by calculating the mean differences (MDs) between groups. If trials have measured the same outcome using different methods, standardized mean differences (SMDs) will be estimated and reported with 95% confidence intervals (CI). For dichotomous outcomes, the effectiveness of the interventions will be summarized as odds ratio (OR) or risk ratio (RR) and reported with 95% CI and standard deviations (SDs).

We anticipate the analysis will include a pairwise meta-analysis and Bayesian network meta-analysis (NMA). Traditional pairwise meta-analysis will be conducted using Review manager V.5.4 and network meta-analysis will be conducted using R packages. The I^2^ statistic will be used to assess the level of heterogeneity of each pairwise comparison. If I^2^ < 50%, a fixed-effect model will be used; otherwise, a random-effect model will be employed. Posterior mean residual deviance (PMRD) will be used to assess the goodness of fit of each model to the data. PMRD is defined as the difference between the deviance of the fitted model and the saturated model. These models will be compared using the deviance information criterion (DIC), calculated by summing the PMRD and the number of effective parameters.

We will rank the relative effects of each treatment in the ranking table. In this ranking table, we will report above the leading diagonal pairwise effect size comparisons, below the leading diagonal effect size estimated from network meta-analyses. Moreover, high odds in P score or surface under the cumulative ranking curve (SUCRA) will provide better results for an intervention [18].

We will also conduct subgroup analyses and meta-regression network analyses to explore statistical heterogeneity among the trials and inconsistency between direct and indirect evidence. We also aim to consider following possible effect modifiers: age and gender of the participant, type of revascularization, duration of intervention, model of intervention, and others.

### 3.1. Geometry of the Evidence Network

The network diagram will graphically represent the evidence derived from multiple interventions to provide the volume of evidence behind each comparison, as well as possible pictorial comparisons where ≥2 interventions are connected to the network and can be compared. The nodes and edges of the network will be used to reveal the head-to-head comparisons among interventions.

### 3.2. Confidence in Cumulative Evidence

We will use appropriate tools to evaluate and synthesize the quality of the evidence across the included trials. This will be conducted using Grades of Recommendations, Assessment, Development, and Evolution (GRADE) [19] and Confidence in Network Meta-analysis (CINeMA) [20].

## 4. Discussion

There has been an increasing interest in preventive cardiology and cardiac rehabilitation along with successful revascularization procedures for patients with CAD. Cardiac rehabilitation comprises multiple core components including risk factor assessment and control, physical activity counseling, exercise training, nutritional counseling, and psychological counseling. EBCR remains at the center of cardiac rehabilitation approaches to manage patients with CAD. EBCR has been shown to improve exercise capacities, reduce the occurrence of cardiac events, reduce mortality, and reduce readmissions in patients with CAD [4,10,21]. Exercise prescription in EBCR has followed established guidelines for aerobic and resistance training. It has also been observed that the reduction in the mortality and hospital admissions was similar in the center-based CR (CBCR) and home-based CR programs (HBCR) [4]. Additionally, according to a recent systematic review and meta-analysis, it has been observed that HBCR using wearable devices has shown to improve exercise capacity when compared to CBCR [22]. However, in clinical practice, often there arises a conundrum of concerns that affect exercise prescription. Therefore, deciding the appropriate intervention and choosing the prescription that is likely to yield the best result is attributed to a clinician’s acumen and experience. A recent review [23] identified limited data on dose-responses in CR, and another systematic review [24] identified variations in the actual exercise prescriptions, albeit from a limited number of studies. This review aims to address these uncertainties and fill the gap in the literature.

In settings where there are insufficient head-to-head comparisons of different interventions and dosages on exercise capacity improvements, the need for the clinician to have access to evidence to support clinical decision-making is paramount. The standard pair-wise meta-analysis generates one pool estimate, whereas a NMA will generate more than one pool estimate. This NMA will assemble and summarize all the exercise-based interventions from published RCTs in patients with CAD. Specifically, this NMA will return the ranking of the various exercise modalities, which will determine the most and the least effective interventions in this population. Such information will be important to guide the consulting physician and cardiac rehabilitation specialists to prescribe the most appropriate and beneficial interventions.

Considering the methodological rigor of the review, we feel the study would be relatively free from bias. However, we must be open to some selection bias, as we are assessing only studies in the English language and only those indexed.

## 5. Conclusions

To conclude, this protocol has laid the foundation for a full systematic review and NMA which will help provide vital information to the EBCR professional to guide exercise prescription in those with CAD. It will also form a foundation to guide future head-to-head trials comparing different interventions and will, thereby, build the EBCR literature database.

## Figures and Tables

**Table 1:** PICO for the search strategy.

Population (P)	Those with coronary artery disease
Intervention (I)	Exercise based cardiac rehabilitation
Comparator (C)	Controls or another exercise-based intervention
Outcome (O)	Exercise capacity

**Table 2:** Search String used in PubMed.

No.	Search Terms
#1	“coronary artery disease”
#2	“coronary artery disease” [Title/Abstract}
#3	Coronary artery disease [MeSH Terms]
#4	Coronary artery bypass [MeSH Terms]
#5	Angioplasty, percutaneous transluminal [MeSH)
#6	#1 OR #2 OR #3 OR #4 OR #5
#7	Aerobic exercise [MeSH Terms]
#8	Breathing exercise [MeSH Term]
#9	Resistance training [MeSh Terms]
#10	Exercise *
#11	“Cardiac rehabilitation”
#12	“inspiratory muscle training”
#13	“respiratory muscle training”
#14	“high intensity interval training”
#15	HIIT
#16	#7 OR #8 OR #9 OR #10 OR #11 OR #12 OR #13 OR #14 OR #15
#15	#4 AND #16

**Table 3:** Description of the exercise training interventions.

Intervention	Brief Description
Aerobic exercises	This includes any physical activity that uses large muscle groups, and that is continuous and rhythmic in nature
Strength/Resistance training	This is a form of physical training that is designed to improve muscular strength and endurance by exercising a muscle or a muscle group against external resistance
High-intensity interval training (HIIT)	Form of interval training with alternating short bouts of vigorous to high-intensity exercise interspersed with moderate-intensity recovery periods
Inspiratory muscle training (IMT)	IMT is a form of strength training which involves breathing exercises using a variety of devices that strengthens the inspiratory muscles of respiration
Combination	This involves a combination of ≥2 of the above interventions

## Data Availability

Data will be available with the lead and corresponding authors and will be made available on reasonable requests.

## Supplementary Materials

The following supporting information can be downloaded at: https://www.mdpi.com/article/10.3390/ijerph191912317/s1, Supplement Table S1: Search string.
