# Supplementary material for: Identifying the Optimal Exercise Prescription for Patients with Coronary Artery Disease Undergoing Cardiac Rehabilitation: Protocol for a Systematic Review and Network Meta-Analysis of Randomized Control Trials
Source: Int J Environ Res Public Health. 2022 Sep 28;19(19):12317. doi: 10.3390/ijerph191912317 (PMC9566022; doi:10.3390/ijerph191912317)
Supplement: Supplementary file 1 [file ijerph-19-12317-s001.zip › ijerph-1913558-supplementary.pdf]

Table S1. Search strategy for individual databases

CINAHL

#1	Aerobic exercise
#2	Aerobic training
#3	Resistance training
#4	Strength training
#5	Weight training
#6	HIIT
#7	High intensity training
#8	High intensity interval training
#9	Inspiratory muscle training
#10	Respiratory muscle training
#11	Respiratory muscle strength training
#12	Cardiac rehabilitation
#13	#1 OR #2 OR #3 OR #4 OR #5 OR #6 OR #7 OR #8 OR #9 OR #10 OR #11 OR #12
#14	Coronary artery disease

#15	CAD
#16	Coronary heart disease
#17	CHD
#18	Ischemic heart disease
#19	Ischaemic heart disease
#20	Coronary artery bypass graft
#21	CABG
#22	PCI
#23	Percutaneous coronary intervention
#24	#14 OR #15 OR #16 OR #17 OR #18 OR #19 OR #20 OR #21 OR #22 OR #23
# 25	#13 AND #24

EMBASE

#1	Aerobic exercise
#2	Aerobic training
#3	Resistance training
#4	Strength training

#5	Weight training
#6	HIIT
#7	High intensity training
#8	High intensity interval training
#9	Inspiratory muscle training
#10	Respiratory muscle training
#11	Respiratory muscle strength training
#12	Cardiac rehabilitation
#13	#1 OR #2 OR #3 OR #4 OR #5 OR #6 OR #7 OR #8 OR #9 OR #10 OR #11 OR #12
#14	Coronary artery disease
#15	CAD
#16	Coronary heart disease
#17	CHD
#18	Ischemic heart disease
#19	Ischaemic heart disease
#20	Coronary artery bypass graft
#21	CABG

#22	PCI
#23	Percutaneous coronary intervention
#24	#14 OR #15 OR #16 OR #17 OR #18 OR #19 OR #20 OR #21 OR #22 OR #23
# 25	#13 AND #24

Web of Science

#1	Aerobic exercise
#2	Aerobic training
#3	Resistance training
#4	Strength training
#5	Weight training
#6	HIIT
#7	High intensity training
#8	High intensity interval training
#9	Inspiratory muscle training
#10	Respiratory muscle training
#11	Respiratory muscle strength training

#12	Cardiac rehabilitation
#13	#1 OR #2 OR #3 OR #4 OR #5 OR #6 OR #7 OR #8 OR #9 OR #10 OR #11 OR #12
#14	Coronary artery disease
#15	CAD
#16	Coronary heart disease
#17	CHD
#18	Ischemic heart disease
#19	Ischaemic heart disease
#20	Coronary artery bypass graft
#21	CABG
#22	PCI
#23	Percutaneous coronary intervention
#24	#14 OR #15 OR #16 OR #17 OR #18 OR #19 OR #20 OR #21 OR #22 OR #23
# 25	#13 AND #24
